# The Role of Eryptosis in the Pathogenesis of Renal Anemia: Insights From Basic Research and Mathematical Modeling

**DOI:** 10.3389/fcell.2020.598148

**Published:** 2020-12-09

**Authors:** Gabriela Ferreira Dias, Nadja Grobe, Sabrina Rogg, David J. Jörg, Roberto Pecoits-Filho, Andréa Novais Moreno-Amaral, Peter Kotanko

**Affiliations:** ^1^Graduate Program in Health Sciences, Pontifícia Universidade Católica do Paraná, Curitiba, Brazil; ^2^Renal Research Institute, New York, NY, United States; ^3^Fresenius Medical Care Deutschland GmbH, Bad Homburg, Germany; ^4^Arbor Research Collaborative for Health, Ann Arbor, MI, United States; ^5^Icahn School of Medicine at Mount Sinai, New York, NY, United States

**Keywords:** kidney failure, anemia, eryptosis, erythropoietin, phosphatidylserine, calcium, hypoxia, oxidative stress

## Abstract

Red blood cells (RBC) are the most abundant cells in the blood. Despite powerful defense systems against chemical and mechanical stressors, their life span is limited to about 120 days in healthy humans and further shortened in patients with kidney failure. Changes in the cell membrane potential and cation permeability trigger a cascade of events that lead to exposure of phosphatidylserine on the outer leaflet of the RBC membrane. The translocation of phosphatidylserine is an important step in a process that eventually results in eryptosis, the programmed death of an RBC. The regulation of eryptosis is complex and involves several cellular pathways, such as the regulation of non-selective cation channels. Increased cytosolic calcium concentration results in scramblase and floppase activation, exposing phosphatidylserine on the cell surface, leading to early clearance of RBCs from the circulation by phagocytic cells. While eryptosis is physiologically meaningful to recycle iron and other RBC constituents in healthy subjects, it is augmented under pathological conditions, such as kidney failure. In chronic kidney disease (CKD) patients, the number of eryptotic RBC is significantly increased, resulting in a shortened RBC life span that further compounds renal anemia. In CKD patients, uremic toxins, oxidative stress, hypoxemia, and inflammation contribute to the increased eryptosis rate. Eryptosis may have an impact on renal anemia, and depending on the degree of shortened RBC life span, the administration of erythropoiesis-stimulating agents is often insufficient to attain desired hemoglobin target levels. The goal of this review is to indicate the importance of eryptosis as a process closely related to life span reduction, aggravating renal anemia.

## Introduction

Red blood cells (RBCs) are vital to life, and their oxygen carrying role is indispensable to the function of tissues and organs. In healthy humans, RBCs undergo senescence and cell death after around 120 days. RBCs can also undergo a distinct mechanism of death, a process of programmed RBC death similar to apoptosis, called eryptosis (Qadri et al., 2017). This may occur throughout the RBC lifetime, even before senescence under various stress conditions, and it is increased in kidney failure patients for reasons only partially understood (Abed et al., 2014; Bissinger et al., 2016; Dias et al., 2018). The RBC plasma membrane acts as a selective barrier that ensures a constant internal composition, by controlling the active and passive transfer of ions and molecules. Composed of more than 50 transmembrane proteins, the membrane regulates RBC shape, as well as mobility, deformability, and ion and macromolecules transport (Mohandas and Gallagher, 2008). Membrane proteins play an important role in regulating RBC volume by controlling the movement of ions and thus assure cell deformability while traversing capillaries and spleen sinusoids (Gallagher, 2013; Glogowska and Gallagher, 2015). Fluidity of the cytoplasm and cell volume regulation are necessary for capillary transit and transport of O_2_ and CO_2_ (Narla and Mohandas, 2017). Beyond their primary O_2_-carrying function, RBCs are essential for systemic metabolic processes, such as pH regulation, nitric oxide production, and immune responses (Nemkov et al., 2018; Rifkind et al., 2018).

The capability to perform these functions decreases as RBCs approach senescence. In healthy subjects, a delicate balance between RBC death and production rates is maintained, resulting in stable RBC counts in the peripheral blood.

In kidney failure, the erythropoiesis rate is reduced, leading to fewer circulating RBCs. It is well established that the main cause of renal anemia is an inadequately low renal erythropoietin (Epo) synthesis combined with functional iron deficiency. Additionally, kidney patients show a dysregulation of oxygen sensing via the hypoxia-inducible factor pathway (Guedes et al., 2020). Erythropoiesis-stimulating agents (ESAs) are routinely used to compensate for the shortfall in endogenous Epo production. However, hypo-responsiveness to ESA is seen in 5–10% of anemic CKD patients. These patients do not achieve prescribed hemoglobin targets despite high ESA doses. This can be partially explained by accentuated inflammation and iron deficiency (Ogawa and Nitta, 2015). A less recognized potential cause of ESA hypo-responsiveness is decreased RBC life span. Eryptosis may reduce RBC survival and contribute to renal anemia since in some patients the erythropoiesis rate cannot compensate for this increased loss (Lang et al., 2017). In this review, we describe the role of eryptosis in the pathogenesis of renal anemia, an aspect frequently neglected in the clinical practice.

## The Pathogenesis of Renal Anemia: The Epo-Centric View

Most chronic kidney disease (CKD) patients suffer from anemia at some point in the course of their illness. Epo levels in CKD patients are well below those seen in anemic non-kidney failure patients at the same level of hematocrit. The first cases of patients with renal anemia treated with ESA showed a dramatic effect: A few days after initiation of ESA therapy, their hematocrit approached normal levels, necessitating a reduction in ESA dose. The marked increase in RBC mass following treatment with ESA was accompanied by enhanced utilization of iron stores, as reflected in a decline in serum iron and serum ferritin (Eschbach et al., 1987; Bunn, 2013). To many of those who witnessed these first results, the challenges of treating renal anemia may have become a matter of the past. However, decades later, many open questions have remained.

## Eryptosis Pathways: Overview

Anemia is considered as a non-conventional risk factor in patients with CKD, especially in those on dialysis (Iseki and Kohagura, 2007). As discussed above, renal anemia is mainly attributed to decreased production of Epo by diseased kidneys, compromising erythropoiesis. In addition, several studies indicate that eryptosis is increased in CKD patients leading to early and accelerated elimination of circulating RBCs (Abed et al., 2014; Bissinger et al., 2016; Bonan et al., 2016; Dias et al., 2018).

Eryptosis is characterized by RBCs undergoing morphologic changes such as cell shrinkage, membrane scrambling, and the exposure of phosphatidylserine (PS) (Lang and Qadri, 2012). These changes are stimulated by Ca^2+^ influx into the RBCs through non-selective Ca^2+^ channels, which in turn can be activated by prostaglandin E2 (PGE2) formation, oxidative and osmotic stress, as well as Cl^–^ efflux (Lang et al., 2007). Activation of Ca^2+^ channels results in an increase of cytosolic Ca^2+^, which can further induce floppase to expose PS on the cell surface and subsequent recognition, engulfment, and degradation of RBCs by macrophages (Lang et al., 2012) and pro-inflammatory monocytes (Bonan et al., 2016). Ca^2+^ may also stimulate sphingomyelinase to form ceramide, which in turn activates scramblase and culminates in loss of asymmetry of the RBC cell membrane and PS exposure (Lang et al., 2010).

Although eryptosis and senescence result in RBC death and clearance from circulation, the mechanisms driven by each pathway differ considerably. While eryptosis is orchestrated by the mechanisms mentioned above, the removal of aged RBCs relies mainly on the reduced deformability of the cells and macrophage recognition of immunoglobulin G and complement factor 3 on the surface of the senescent RBC. Externalization of PS was shown to be negligible in old RBCs (Franco et al., 2013). However, this RBC population is more susceptible to eryptosis induced by energy depletion (Ghashghaeinia et al., 2012). Thus, PS exposure seems to be more relevant for eryptosis rather than RBC senescence (Qadri et al., 2017).

Eryptosis is considered a useful mechanism to avoid a potentially fatal complication of hemolysis, by starting a cell death program with controlled removal before any damage can cause uncontrolled hemolysis (Föller et al., 2008). Since most of the iron content in the body is bound to hemoglobin, phagocytosis and the degradation of RBCs represent an important source of iron. The amount of recycled iron is sufficient to maintain the daily iron requirement for erythropoiesis (Ginzburg and Li, 2010). However, excessive eryptosis can compromise microcirculation through the adhesion of RBCs exposing PS to endothelial receptors of the vascular wall (Borst et al., 2012) and lead to anemia due to the exacerbated RBC clearance by the immune system (Bonomini et al., 2001; Bonan et al., 2016). Enhanced eryptosis has been observed in some clinical conditions, such as diabetes, uremic hemolytic syndrome, sepsis, sickle cell anemia, and CKD, among others (Lang and Lang, 2015). PS exposure was observed to be significantly increased in RBCs from patients undergoing hemodialysis (HD) compared to RBCs from healthy individuals (Abed et al., 2014; Bissinger et al., 2016; Dias et al., 2018). Also, PS exposure was significantly higher in patients on peritoneal dialysis (PD) compared with HD patients (Bissinger et al., 2016). In PD patients, the residual glomerular filtration rate was inversely correlated with percentage of eryptosis. This correlation might be explained by a better clearance of retention solutes in patients with residual kidney function (Virzì et al., 2019). The question whether the HD therapy ameliorates or triggers eryptosis remains controversial. Results suggesting both an increase (Abed et al., 2014) and a reduction (Meyring-Wösten et al., 2017) of PS exposure post HD session were reported.

Eryptosis can be triggered by a range of both endogenous and exogenous insults, including toxins, drugs, and acute and chronic diseases (Lang and Lang, 2015). Among the uremic solutes that accumulate in CKD, acrolein (Ahmed et al., 2013b), methylglyoxal (Nicolay et al., 2006), and indoxyl sulfate (IS) (Ahmed et al., 2013a; Dias et al., 2018; Tozoni et al., 2019) were shown to increase eryptosis. Moreover, stressors including osmotic shifts, oxidative stress, and energy depletion may also contribute to a shortened RBC survival (Lang et al., 2006).

A reversion of PS exposure was shown by the addition of the antioxidant *N*-acetyl-L-cysteine to senescent RBCs (Ghashghaeinia et al., 2012) and incubation of uremic RBCs in healthy plasma (Bonomini et al., 1999). Also, the PS exposure induced by IS in healthy RBCs was attenuated by diphenyleneiodonium chloride (an NADPH oxidase inhibitor) and by ketoprofen (an organic anion transporter 2 inhibitor) (Dias et al., 2018).

## RBC Microvesicles Release

Microvesicle (MV) release is part of the physiological RBC aging process *in vivo*, which indicates a disruption of the network between the lipid bilayer and the cytoskeleton. Moreover, the presence of PS on the surface of MV allow for their recognition by the immune system (Burnier et al., 2009; Leal et al., 2018). The addition of Ca^2+^ to RBC media promotes MV release (Nguyen et al., 2016). This finding suggests the participation of MV in eryptosis when RBC Ca^2+^ is increased. However, the vesiculation process in eryptosis is still poorly understood.

The involvement of PS translocation in MV generation remains controversial. Some studies claim that MV release occurs independent of PS (Williamson et al., 1992; Bucki et al., 1998). Conversely, other authors reported that scramblase inhibition reduced MV release from Ca^2+^-stimulated RBCs, suggesting the participation of PS translocation (Gonzalez et al., 2009). In contrast to cells undergoing apoptosis, RBC form MV from the plasma membrane with minute loss of the lipid order, possibly due to the absence of intracellular organelle membranes (Pyrshev et al., 2018).

MV release was shown to be increased in RBCs from HD patients and attributed to an impacted membrane–cytoskeleton interaction, such as the proteolytic breakdown of band 3 (Antonelou et al., 2011). The uremic solutes IS and indol acetic acid (IAA) induced PS exposure and MV release from healthy RBCs (Gao et al., 2015). PS inhibition with lactadherin reduced MV release, reinforcing the participation of PS and micro-vesiculation in eryptosis. The authors also implicated MV release from RBC in thrombus formation, which may aggravate cardiovascular events in CKD (Gao et al., 2015).

## The Role of Iron in Anemia and Eryptosis

Another relevant aspect of renal anemia is the functional iron deficiency due to increased iron storage in the reticuloendothelial system. In addition, increased hepcidin levels are frequently observed in CKD patients, resulting in poor intestinal iron absorption. CKD patients are also prone to iron loss from (micro)bleeds and iatrogenic causes, such as frequent blood draws and blood loss in the extracorporeal system of dialysis machines. Poor iron availability contributes to impaired eryptosis in concert with the elevated levels of pro-inflammatory cytokines and hepcidin. Hence, ESA therapy is commonly accompanied by iron supplementation (Wish et al., 2018). RBCs from mice fed with an iron-deficient diet showed an increased Ca^2+^ uptake, RBC PS exposure, and eryptosis. Eryptotic RBCs were rapidly cleared from the circulation and thus may have amplified iron deficiency (Kempe et al., 2006). However, excessive iron administration may result in iron store pathologies driven by intracellular iron accumulation. As a consequence of inflammation and reticuloendothelial blockade of iron release, patients might still experience low erythropoiesis rate despite increased iron content (Wish, 2006). RBCs from patients with hemochromatosis showed increased PS exposure, mostly as a result of oxidative stress (Du Plooy et al., 2018).

## The Role of Reactive Oxygen Species in Eryptosis

Patients with CKD, especially when on dialysis, are exposed to a variety of stimuli that change RBC number and phenotype. A critical contributor to eryptosis in CKD is the enhanced oxidative stress. The overproduction of pro-oxidant molecules in CKD is multifactorial and HD itself can activate inflammatory responses; in addition, essential antioxidants, such as vitamins, may be cleared by HD (Bissinger et al., 2018). Oxidative stress is classically defined as the imbalance between pro-oxidants and antioxidants in favor of the former. Oxidative stress exerts its detrimental effects through oxidation of macromolecules. However, it is now clear that oxidative stress is a compartmentalized event that occurs at different levels, from cellular compartments to cells to the whole organism (Santolini et al., 2019). A more recent definition of oxidative stress indicates the importance of a disruption of redox signaling and control and/or molecular damage (Jones, 2006). This broader definition recognizes that damage to macromolecules is not the only pathway by which oxidative stress promotes disease. In fact, changes in cell signaling mediated by ROS can develop in a series of alterations and affect the body in a pathway- and organ-specific manner (Jones, 2006; Halliwell and Gutteridge, 2015).

Both enzymatic and non-enzymatic antioxidant systems are altered in CKD patients (Ling and Kuo, 2018). The thiol glutathione (GSH) is important for the maintenance of RBC redox homeostasis, where it is present at higher concentrations in the cytosol (Valko et al., 2007). This powerful redox buffer system provides an overall picture of the organism’s redox state (Jones, 2006). In one study, the GSH concentration in RBCs from HD patients and healthy subjects was similar while the ratio of GSH and its oxidized form, glutathione disulfide (GSSG), was 40% lower (Khazim et al., 2013). We recently found that the GSH content in RBCs from HD patients is halved compared to RBCs from healthy subjects (Dias et al., 2018); however, the fact that the control subjects were significantly younger may have impacted the results since RBCs from elderly individuals tend to have lower GSH levels (Lupescu et al., 2015). Consistent with this finding, previous studies in uremic and HD patients reported low activity of γ-glutamylcysteine synthetase, a key enzyme in GSH biosynthesis (Alhamdani, 2005). Antioxidant enzymes, such as glutathione peroxidase, which detoxifies hydrogen peroxide (H_2_O_2_), also show a reduced activity in RBCs and plasma of uremic patients (Zachara et al., 2004). Although HD partially increased plasma antioxidant enzyme activities immediately after treatment, their function is not completely restored compared to healthy controls (El-Far et al., 2005).

Different from their non-uremic counterparts, uremic RBCs show activated non-selective Ca^2+^ transporters and subsequent increased Ca^2+^ influx, which triggers eryptosis (Abed et al., 2014). Oxidative stress participates in this process by maintaining Ca^2+^ levels high through the inhibition of the enzyme Ca-ATPase (Mohanty et al., 2014). Autoxidation of hemoglobin is the main pathway of free radical production in RBCs, leading to anion superoxide formation (Çimen, 2008). It was observed that RBCs can release H_2_O_2_ (Huertas et al., 2013; Rifkind et al., 2018). Under hypoxic conditions, RBCs increased superoxide formation and further dismutation to H_2_O_2_. The latter was then diffused from the RBCs and promoted inflammation in the lung microvascular endothelium (Kiefmann et al., 2008). Given the high concentration of hemoglobin in the blood, it is conceivable that even a minor increase of hemoglobin autoxidation could trigger an imbalanced redox state, especially in a population with an already defective antioxidant system, such as CKD.

Moreover, iron facilitates redox reactions, and its accumulation leads to the generation of hydroxyl radical, a powerful ROS, via Fenton’s reaction (Nakanishi et al., 2019). Ferritin levels not only reflect body iron stores but also serve as a biomarker of inflammation. Ferritin and oxidative stress markers are correlated, possibly due to an increased dissociation of iron from ferritin. An increase in unbound iron catalyzes oxidative reactions and promotes cell damage (Kell, 2009; Kell and Pretorius, 2014). The addition of physiological iron levels results in slight RBC shape changes (Pretorius, 2013). Scanning electron microscopy showed that generation of hydroxyl radicals induced by iron overload triggers the aggregation of RBC with fibrin-like fibers, resulting in a pro-thrombotic state (Lipinski et al., 2012).

Despite substantial evidence regarding oxidative biomarkers in CKD, some findings remain controversial and their significance is unclear (Tucker et al., 2013). The high levels of pro-oxidants in tandem with the defective antioxidant machinery found in CKD may contribute to oxidative stress, an enhanced susceptibility of RBCs for eryptosis and worsening of renal anemia.

## Oxidative Stress, Inflammation, and Aging

The triad of aging, inflammation, and oxidative stress reduces the quality of life of CKD patients. The emblematic term *inflammaging* has been introduced to highlight the role of these factors in the often rapid deterioration of the patient’s health, longevity, and well-being (Ebert et al., 2020). Interestingly, inflammatory biomarkers such as interleukin 6 and C-reactive protein are not correlated with RBC life span in HD patients (Ma et al., 2017). Aging and oxidative stress are processes that can take place at the cellular level or the whole-body level and aging is associated with an imbalanced redox environment and rise in inflammatory biomarkers (Maurya et al., 2015).

**FIGURE 1:**
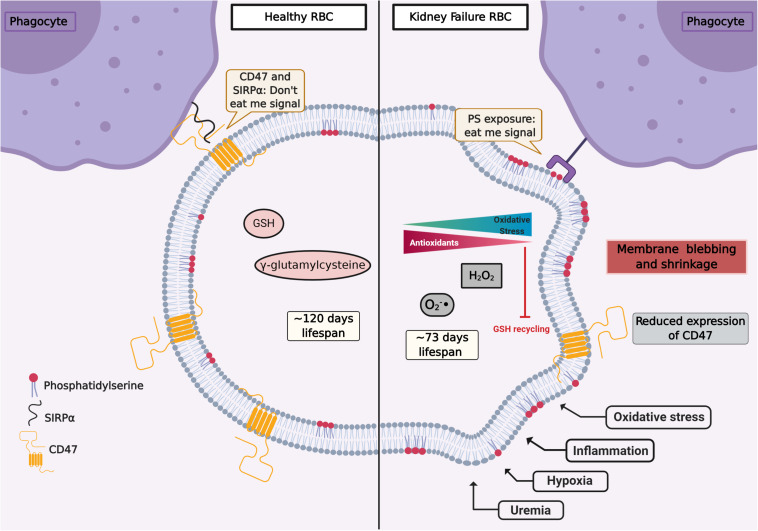
Representation of RBC signaling, life span, and redox environment in health (left) and kidney failure (right). Several factors such as uremia, hypoxia, inflammation, and oxidative stress can induce signals of cellular damage and instigate eryptosis. CD47 is a constitutive transmembrane protein that interacts with receptors on the surface of phagocytes, preventing erythrophagocytosis (“don’t eat me signal”). In health, after about 120 days, CD47 expression is reduced and RBCs are removed by the monocytic–macrophage system, especially in the spleen. The RBC life span is reduced in chronic kidney disease (CKD). RBCs from CKD patients show an overproduction of oxidants and an impaired antioxidant recycling. In CKD, circulating RBCs increase the translocation of phosphatidylserine (PS) from the inner membrane leaflet to the cell surface. Pro-inflammatory monocytes and resident macrophages recognize PS, and RBCs are cleared from circulation. Figure created with BioRender.com.

The oxidative injury during the RBC life span may lead to dysfunction and cell death. Although oxidative stress is fundamental to RBC senescence, it is not the sole reason for its progression. CD47, a constitutive membrane receptor, acts as a protective (“do not eat me”) signal against phagocytosis by interacting with the macrophage receptor SIRPα. Conversely, the expression of PS on RBC surface has the opposite effect and acts as an “eat me” signal to phagocytic cells (Lutz and Bogdanova, 2013; Arias and Arias, 2017; Figure 1). Moreover, Burger et al. found that aging promoted by oxidative stress induces a conformational change of CD47, which enables this molecule to bind to thrombospondin-1, converting CD47 into an “eat me” signal (Burger et al., 2012). The role of O_2_ is highlighted by the observation that RBCs from male subjects performing hypoxic exercise training (15% O_2_) showed less CD47 expression as well as a reduction of the cytoskeleton proteins actin and spectrin. RBC deformability, which is essential for their physiological function, was compromised in hypoxic conditions by the activation of Gardos channel (Mao et al., 2011). Moreover, in RBCs from CKD patients, a reduced expression of CD47 was observed (Antonelou et al., 2011). These different mechanisms underlying RBC senescence are still subject of ongoing research.

From an inflammation perspective, there are several molecules that play a role in renal anemia. The lipid peroxidation product 4-hydroxy-trans-2-nonenal (HNE) contributes to several inflammatory and degenerative processes. It is overexpressed in kidneys from aged rats and leads to NF-κB activation (Jang et al., 2016). In RBCs, HNE exerts a pro-eryptotic effect, initiating the classical eryptotic markers as well as agglutination elements and adhesion molecules, resulting in binding of RBCs to endothelial cells, possibly promoting thrombosis (Allegra et al., 2020).

## Hypoxia in Dialysis Patients and Its Association With Eryptosis

**FIGURE 2:**
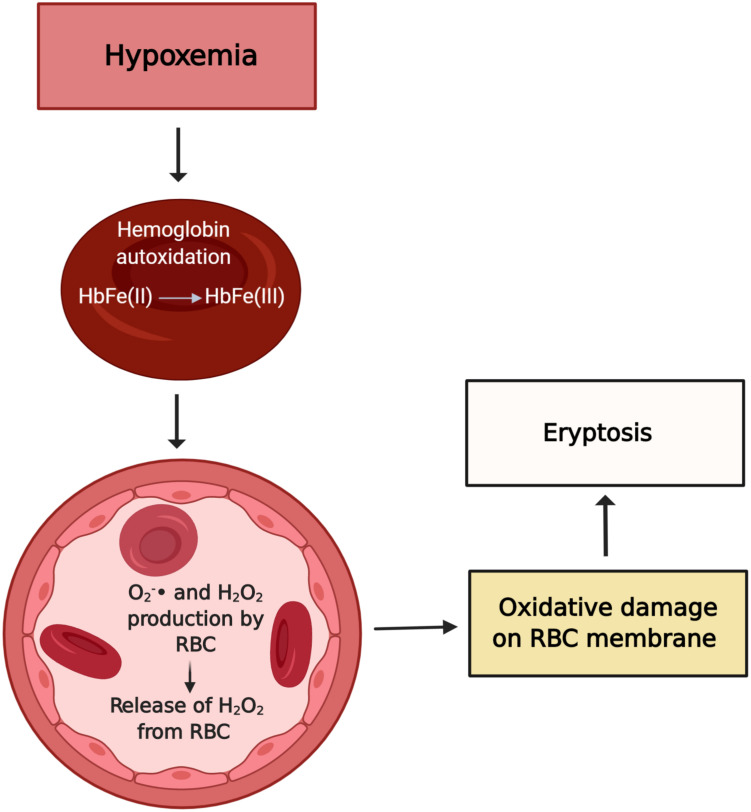
Possible effects of hypoxemia on RBC. Hypoxia leads to hemoglobin autoxidation and formation of O_2_^–^ and H_2_O_2_. In CKD, a weakened antioxidant system fails to prevent injury by oxidants. The exacerbated oxidative stress is a stimulus to eryptosis. Figure created with BioRender.com.

Hypoxia *per se* is well known to provoke oxidative stress. In the early 1990s, Rifkind et al. (1991) showed that the hypoxemic condition facilitates hemoglobin autoxidation and, as a result, the free radical anion superoxide is exacerbated despite the high levels of antioxidants normally present in RBC. Importantly, about 10% of patients undergoing HD experience prolonged intradialytic hypoxemia (PIH), a clinical phenotype characterized by an arterial oxygen saturation below 90% for at least one third of the dialysis treatment time (Meyring-Wosten et al., 2016). Previously, we explored the effect of low oxygen partial pressure and the uremic toxin IS on eryptosis (Tozoni et al., 2019). Interestingly, we found that hypoxemia and IS independently increase eryptosis and ROS generation and decrease GSH levels, possibly contributing to the reduced RBC life span observed in CKD (Figures 1, 2). Of note, high altitude, another hypoxia model, had a different effect on RBC homeostasis. Epo produced in response to hypoxia inhibits Ca^2+^ channels and thus attenuated eryptosis (Myssina et al., 2003). In rats kept at high altitude (5,000 m) for 30 days, chronic hypoxia inhibited eryptosis, possibly by increasing CD47 expression and decreasing intracellular Ca^2+^ levels (Tang et al., 2018). This observation is possibly caused by the protective effect of Epo and production of new RBC with high CD47 expression.

Any harmful insult to RBC will generate membrane modifications and possibly signal an eryptotic event, reversible or not (Pretorius et al., 2016a). Raman spectroscopy reveals several changes in hemoglobin morphology and function of RBCs under hypoxia. According to Revin et al. (2017), not only the lipid composition of RBC is profoundly altered in hypoxia, but also the ability of hemoglobin to bind and select ligands is reduced, including its affinity for oxygen (Chowdhury and Dasgupta, 2017). Morphological modifications of RBCs in tandem with low hemoglobin concentration can result in poor O_2_-carrying capacity and compound tissue hypoxia.

## Ion Channel Modifications in Eryptosis

The disturbance of membrane asymmetry that favors PS exposure and subsequent eryptosis is caused by the increase of cytosolic Ca^2+^ (Segawa and Nagata, 2015; Pretorius et al., 2016a). Ca^2+^ enters RBC through several ion channels, pumps, and exchangers that transport Ca^2+^ through the plasma membrane (Polak-Jonkisz et al., 2010b; Brini and Carafoli, 2011; Polak-Jonkisz and Purzyc, 2012). Several factors such as oxidative stress, energy depletion, or uremic toxins activate Ca^2+^ influx into the RBC (Lang and Lang, 2015). Ca^2+^ efflux occurs mainly by a high-affinity, low-capacity Ca^2+^-ATPase, the plasma membrane Ca^2+^ pump (PMCA) (Brini and Carafoli, 2011).

**FIGURE 3:**
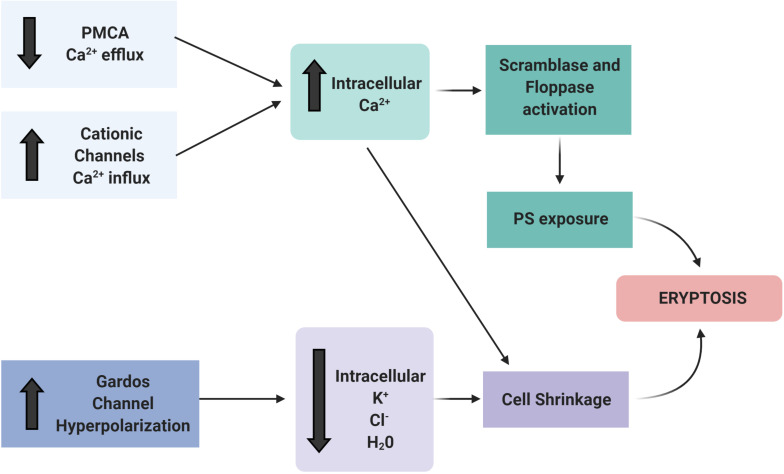
Ion channels involved in eryptosis. The activation of Ca^2+^ channels (e.g., by uremic toxins, oxidative stress, hypoxia) is the main trigger to initiate the events that culminate in eryptosis. The increased intracellular Ca^2+^ activates scramblase and floppase proteins that promote PS exposure on the cell surface. Patients with CKD have PMCA inactivation and subsequent reduced Ca^2+^ efflux. In addition, other ion channels, such as the Gardos channel, are altered. The resulting efflux of K^+^, Cl^–^, and water result in RBC shrinkage and induce eryptosis. Figure created with BioRender.com.

**FIGURE 4:**
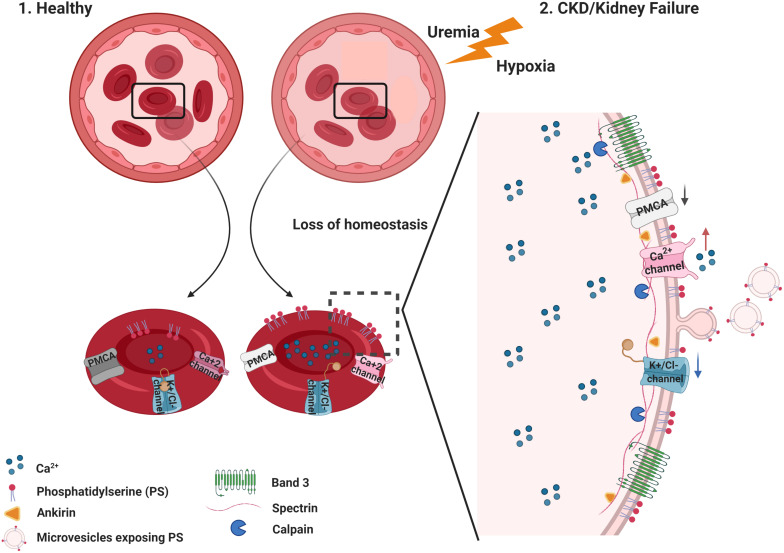
Summary of membrane and cytoskeleton modifications in eryptosis.(1) Healthy RBC maintain normal intracellular levels of Ca^2+^ by active mechanisms controlling its efflux and influx. Under these circumstances, phosphatidylserine (PS) is located in the inner leaflet of the RBC membrane. (2) In CKD, uremic toxins accumulate and low hemoglobin oxygen saturation (hypoxemia) is observed in some patients. This loss of homeostasis favors significant changes in the stability of the RBC, with an increase in the Ca^2+^ influx and, consequently, an increase in its intracellular concentration. Increased intracellular Ca^2+^ trigger PS translocation to the outer leaflet of the RBC. Several processes follow the Ca^2+^ increase, such as the activation of calpains (calcium-activated proteases) that target cytoskeletal proteins such as spectrin, ankyrin, as well as band 3/AE1. These processes result in the degradation of cytoskeletal proteins, microvesicle release, and changes in cell shape and deformability. Figure created with BioRender.com.

The distribution of PS on the inner and outer membrane leaflet is determined by the activity of translocase proteins in the RBC membrane: flippase, floppase, and scramblase. Flippase is an ATP-dependent transporter that transfers phospholipids from the extracellular leaflet to the cytoplasm, while floppase is an ATP-binding cassette transporter that catalyzes the movement of phospholipids in the opposite direction (Hankins et al., 2015). To maintain an optimal distribution of lipid bilayer phospholipids, scramblases regulate PS movement in both directions, independent of ATP. These enzymes are regulated by intracellular Ca^2+^ levels (Bitbol et al., 1987; Pretorius et al., 2016a; Föller and Lang, 2020). A high concentration of cytosolic Ca^2+^ inhibits flippase, which results in the activation of the scramblase, followed by the translocation of PS from the internal leaflet to the surface of the eryptotic RBC (Williamson et al., 1992; Figures 3, 4).

Intracellular Ca^2+^ regulation is mediated mainly by its low passive permeability and its active removal by the calcium ATPase pump dependent on Ca^2+^-Mg^2+^ and by the Na^+^/Ca^2+^ exchanger (Carafoli, 1987; Blaustein, 1988; Reeves et al., 1994). The Ca^2+^ PMCA1 and PMCA4 regulate and maintain the internal concentration of Ca^2+^. About 10% of the plasma RBC membrane proteins are PMCAs (Rothstein et al., 1976). These enzymes are important regulators of Ca^2+^ homeostasis, being activated by a series of mechanisms, some of them though still unknown.

Ca^2+^ efflux from the cytosol is against a steep chemical gradient and hence PMCA requires ATP. RBCs from CKD patients have a decreased PMCA activity as well as reduced calmodulin concentration as renal failure progresses (Polak-Jonkisz et al., 2010a). Thus, elucidating the relationship between the systems that control both Ca^2+^ influx and efflux in eryptosis would be an important step in determining potential inhibitory targets for the accelerated RBC death in CKD.

Although RBC’s PMCA has been well characterized, the knowledge of ion transport systems that mediate Ca^2+^ uptake in RBC is quite limited. The incubation of RBC with Ca^2+^ ionophore ionomycin causes exposure of PS in the outer membrane leaflet (Föller et al., 2009b). In addition to PS exposure, elevated levels of intracellular Ca^2+^ promote oxidative stress by directly activating NADPH oxidase and nitric oxide synthase in uncoupled mode (Özüyaman et al., 2008; George et al., 2013). Among the transport systems that contribute to the uptake of Ca^2+^ in human RBCs, there are several classes of cation channels (Kaestner, 2011). Some ionotropic receptors have been described in RBCs, like the GluA1, the AMP glutamate ionotropic receptor subunit (Makhro et al., 2016; Kaestner et al., 2020), as well as N-methyl-D-aspartate (NMDA) (Makhro et al., 2017), contributing to Ca^2+^ homeostasis in these cells. Moreover, after removal of Cl^–^ or extracellular glucose, alpha-amino-3-hydroxy-5-methyl-4-isoxazolepropionic acid (AMPA) antagonist receptors drive the increase in cytosolic Ca^2+^ and induce eryptosis by stimulating Ca^2+^ influx (Föller et al., 2009a).

Circulating RBCs are exposed to significant mechanical forces that influence their physiology and function in several ways, including deformability, the release of products such as ATP (Sprague et al., 2001), and the Ca^2+^ influx. Ca^2+^ influx also influences cell volume. Changes in RBC volume affect their ability to traverse capillaries. The molecular mechanisms involved in sensing mechanical forces and their effects on RBC volume are not fully understood. The mechanosensitive Ca^2+^ channel PIEZO1 is located on the RBC membrane. PIEZO1 is an important regulator of cell volume in response to mechanical stress (Cahalan et al., 2015). The connection between mechanical forces and RBC volume via Ca^2+^ influx through PIEZO1 is closely linked to the cells’ ability to change shape and reduce cell volume and enable passage through small-diameter capillaries. Of note, the intensity of the mechanical force can induce the initial steps of eryptosis, since an increase of PIEZO1-dependent Ca^2+^ influx stimulates Gardo’s channels and subsequent RBC shrinkage (Cahalan et al., 2015).

Increased Ca^2+^ influx due to Ca^2+^ channel activation combined with reduced Ca^2+^ efflux from the cytosol may affect the homeostasis of other ions, most prominently K^+^. Activation of the Gardos channel, a K^+^ efflux channel activated by intracellular Ca^2+^ increase, results in membrane hyperpolarization and increased Cl^–^ efflux. Finally, the loss of water leads to cell shrinkage (Thomas et al., 2011; Boulet et al., 2018; Figures 3, 4). Even a local membrane deformation can trigger this process and induce cell dehydration, which may explain the higher density of senescent RBCs (Dyrda et al., 2010).

## Cytoskeleton Modifications

The RBC cytoskeleton plays an important role in cell homeostasis. The spectrin-actin network interacts with ankyrin and controls RBC deformability (Pretorius et al., 2016b). This interaction of the cytoplasmatic domain of membrane proteins with cytoskeleton proteins prevents membrane vesiculation and breakup (Mohandas and Gallagher, 2008). Besides the role of Ca^2+^ on the eryptotic process, the prolonged Ca^2+^ permeability activates μ calpain, which can degrade cytoskeleton components, such as the ankyrin R complex that forms bridges to connect membrane proteins to the spectrin-based skeleton, assuring membrane stability and assembly of signaling and structural components on the inner membrane surface (Berg et al., 2001; Figure 4).

The band 3 protein, also called anion exchanger 1 (AE1), is one of the transport proteins that mediate the exchange of Cl^–^ and HCO_3_^–^. Through interaction with lipids and proteins, the multifunctional band 3 unites the multiprotein complex of the cytoskeleton and confers mechanical and elastic properties to RBC and thus blood viscosity (Burton and Bruce, 2011). Moreover, studies of RBC membrane proteins in CKD stage 5 patients showed lower levels of ankyrin and spectrin, as well as altered ankyrin/band 3 ratio. In addition to these alterations, the same study showed that patients in CKD stage 5 who do not respond to ESA have a lower spectrin/ankyrin ratio (Costa et al., 2008).

## RBC Life Span in Health and CKD

Aging RBCs lose the flexibility needed to traverse the network of tight capillaries. Senescent RBCs are removed from circulation by splenic red pulp macrophages. While the key role of Epo deficiency in renal anemia is undisputed, the impact of RBC life span has received only little attention (Dou et al., 2012). One reason is that RBC life span measurements are impractical in the clinical environment. Studies show that the life span of RBC from HD patients is dramatically reduced. Several groups have measured RBC life span in HD patients and arrived, for example, at values of 73 ± 18 days (Ma et al., 2017) and 89 ± 28 days (Sato et al., 2012). While there are rare obvious reasons for hemolysis in dialysis patients (e.g., contaminations with chloramine or nitrate; overheated dialysate) (Saha and Allon, 2017), the pathogenesis of the reduced RBC life span is ill-defined. The shortened RBC life span in CKD has been attributed to the uremic environment rather than mechanical stress induced by HD (Vos et al., 2011). As the RBC life span declines, higher doses of ESAs are needed to attain hemoglobin target levels in HD patients (Sato et al., 2012). Interestingly, ESA administration was positively correlated with PS exposure on RBC from HD and PD patients (Bissinger et al., 2016). On the other hand, eryptosis in healthy RBCs induced by osmotic shock showed the opposite effect, where PS exposure was ameliorated in the presence of Epo (Myssina et al., 2003). These contradictory findings demonstrate that the response of the RBCs to Epo may be dependent on the nature of the eryptotic trigger and the prevailing milieu interieur. Although the administration of ESAs is crucial for the correction of renal anemia, it may promote the clearance of young RBCs, a process called neocytolysis (Alfrey and Fishbane, 2007).

Of note, serum Epo levels do not differ substantially across CKD stages. Interestingly, reticulocyte count was reduced only in CKD stage 5 when compared with stage 1 (Li et al., 2019). Such findings suggest that other factors besides Epo deficiency, such as RBC life span, compound renal anemia. Indeed, the decline of kidney function is correlated with a progressive shortening of RBC life span (122 ± 50, 112 ± 26, 90 ± 32, 88 ± 28, and 60 ± 24 days, from CKD stages 1–5, respectively) (Li et al., 2019), and the prevalence of anemia also increases with the progression of the disease (KDIGO, 2012).

## Relation Between RBC Life Span and Epo Requirement: Insights From Biomathematical Modeling

The higher ESA requirement in patients with a lower RBC life span can also be elucidated using computational models of erythropoiesis. Such models encapsulate key features of human erythropoietic physiology and, through simulations, enable one to study how physiological factors such as RBC life span determine a patient’s response to ESA administrations. Furthermore, they provide a tool to augment the design and interpretation of clinical and laboratory studies and aid the development of treatment algorithms. Phenomenological approaches have focused on an abstract description of the hematocrit as the only model variable, in which ESA administrations lead to an effective increase in hematocrit, without resolving the underlying physiological mechanisms (Uehlinger et al., 1992; Kalicki and Uehlinger, 2008). In this scheme, the duration and progression of the ESA-induced hematocrit increase is determined by RBC life span and its variability, whereas the speed of the increase is determined by effective parameters accounting for ESA efficacy and the concentration threshold for ESA response. Using model simulations, it has been illustrated that a lower RBC life span results in lower hematocrit levels for the same ESA dose (Kalicki and Uehlinger, 2008), consistent with higher ESA doses being required to achieve a desired hematocrit target for lower RBC life span.

**FIGURE 5:**
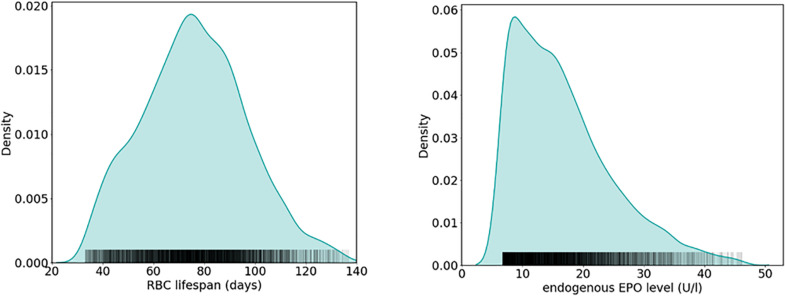
Estimation of physiological parameters in a HD patient population using a physiology-based model of erythropoiesis (Fuertinger et al., 2013). Shown are relative frequencies of estimated RBC life span (left) and endogenous Epo levels (right) obtained from patient-individual model adaptations to hemoglobin and ESA administration data of 6,659 HD patients, resulting in a virtual patient population (Fuertinger et al., 2018b) (see the main text for details).

How does RBC life span affect Epo requirements within an entire patient population, taking into account inter-patient variability in other physiological parameters related to RBC fate as well? Modeling approaches based on “virtual patient populations” can provide insights into this question, as can be illustrated considering an established physiological model of erythropoiesis (Fuertinger et al., 2013, 2018a). This model explicitly represents the proliferative hierarchy of erythroid progenitor populations in the bone marrow including the dynamics of cell birth, maturation, differentiation, and apoptosis; in this modeling scheme, Epo acts as a regulator of apoptosis of the colony-forming unit-erythroid (CFU-E) and erythrocyte populations. Previously, this model has been adapted on a patient-individual level to a large population of HD patients treated with methoxy polyethylene glycol-epoetin beta (6,659 patients randomly sampled from a U.S. HD population comprising over 37,000 patients) (Fuertinger et al., 2018b). These model adaptations were carried out such that previously recorded hemoglobin responses to ESA therapy for the specific patient were described by the model within a predefined accuracy. Individual patients were represented by a patient-specific parameter set (RBC life span, ESA half-life, endogenous Epo levels, and effective parameters accounting for the ESA’s effect on erythroid progenitor apoptosis and maturation velocity) capturing their erythropoiesis-related physiology (Fuertinger et al., 2018b). Although iron availability is not an explicit part of the model, its effects are implicitly present in the effective bone marrow-related parameters. These patient representations through patient-specific parameter sets within the model paradigm are termed “anemia avatars” or “virtual patients.” Estimated mean RBC life span across all virtual patients was 76 ± 21 days (mean ± SD; range: 33–137 days), close to reported values in urban HD centers (see, e.g., Ma et al., 2017; mean ± SD: 73 ± 18 days; range: 38–116 days); the median estimated endogenous Epo level is 15 U/L (25th, 75th percentiles: 10.2 U/L, 21.1 U/L). The relative frequencies of estimated RBC life spans and endogenous Epo levels among the population of virtual patients reported in Fuertinger et al. (2018b) are shown in Figure 5.

**FIGURE 6:**
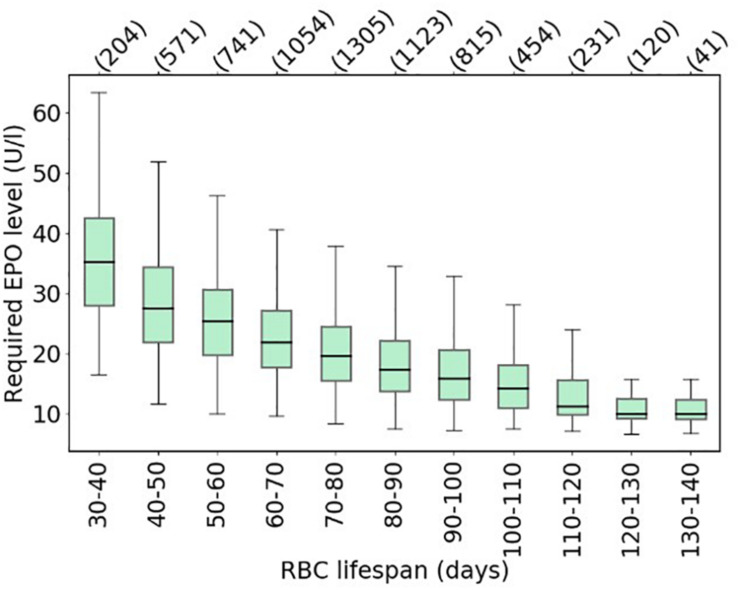
Model-based estimates of the average required Epo levels to achieve a hemoglobin target of 10.49–10.51 g/dl across the virtual patient population shown in Figure 5, binned by patient-specific RBC life span. Boxes show the interquartile range (IQR); whiskers show the full range of values for all virtual patients in the respective bin (bin population indicated in parentheses) excluding outliers defined as being more than ± 1.5 IQR outside the box.

**FIGURE 7:**
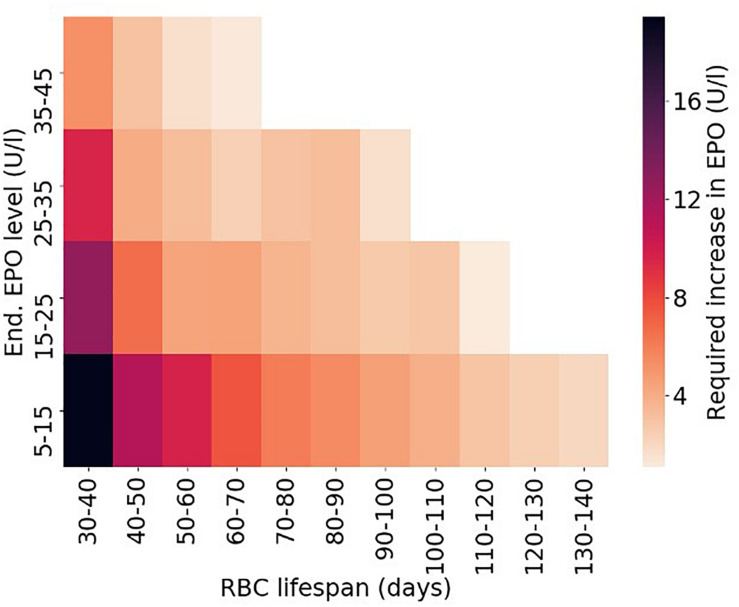
Required average increase in endogenous Epo levels depending on RBC life span and endogenous Epo levels for the virtual patient population shown in Figures 5, 6. Only bins populated with at least 25 virtual patients are included in the density plot.

Making use of this established set of virtual patients, it is straightforward to illustrate how RBC life span affects the amount of Epo required for simulated patients to meet a specific hemoglobin level (10.49–10.51 g/dl). To this end, for each virtual patient, required total Epo levels^1^ are determined. Figure 6 shows the distribution of required Epo serum concentrations to achieve the hemoglobin target, with virtual patients being grouped into RBC life span bins of 10 days. The model analysis suggests a systematic increase in Epo requirement with decreasing RBC life span, a marked trend despite the variability of the virtual patient population in other physiological parameters affecting RBC generation and fate. This inter-patient variability is responsible for the partially large spread of required Epo levels within each binned group, an effect that becomes more prominent for small RBC life spans. Dissecting the virtual patient population by RBC life span and endogenous Epo levels, the mean required increase in Epo concentration to achieve the hemoglobin target is shown in Figure 7: Within the same Epo range, the shorter the RBC life span, the higher the required increase in Epo level, as is most clearly visible for the smallest Epo level range (5–15 U/L). Notwithstanding the effects of ESA half-life, these insights obtained from modeling approaches involving “virtual patient populations” illustrate that (i) a reduced RBC life span may necessitate frequent and high-dose ESA administrations and may present a cause of Epo hypo-responsiveness; (ii) within some (virtual) patients, the effects of a reduced RBC life span on Epo requirements are partially compensated by other physiological factors affecting RBC generation and fate such as endogenous Epo levels.

## Conclusion

Among the many causes that contribute to anemia in kidney failure, eryptosis is a key process whose significance has not been fully acknowledged so far. Although improving hemoglobin levels, ESA and iron administration alone are only one strategy to correct renal anemia that can be limited by a decreased RBC life span. Oxidative stress, inflammation, hypoxemia, and accumulation of uremic solutes promote an imbalance of RBC homeostasis and need to be considered. Even in a scenario where ESA administration increases erythropoiesis rate, the newly formed RBCs can undergo eryptosis within a few days in circulation, resulting in ESA hypo-responsiveness and thus preventing attainment of desired hemoglobin targets. These mechanisms center around (i) increased Ca^2+^ influx and reduced activity of enzymes mediating Ca^2+^ efflux (PMCA), (ii) Gardos channels activation and RBC volume loss, (iii) PS exposure on cell surface and subsequent RBC clearance from circulation. More research in this field is needed to further elucidate these processes and develop potential therapeutic interventions. Extending RBC life span in uremia may evolve as a novel therapeutic strategy for renal anemia.

## Author Contributions

GD and AM-A wrote, reviewed and edited, contributed to the discussion, and created figures. NG wrote, reviewed and edited, and contributed to the discussion. SR wrote, reviewed and edited, contributed to the discussion and carried out the mathematical analysis. DJ and RP-F reviewed, edited, and contributed to the discussion. PK conceptualized, wrote, reviewed and edited, and contributed to the discussion. All authors contributed to the article and approved the submitted version.

## Conflict of Interest

PK holds stock in Fresenius Medical Care. The Renal Research Institute is a wholly owned subsidiary of Fresenius Medical Care. The remaining authors declare that the research was conducted in the absence of any commercial or financial relationships that could be construed as a potential conflict of interest.

## Footnotes

1 Total Epo levels instead of ESA utilization are chosen to eliminate the effects of ESA half-life, which has a high inter-patient variability that would confound the relation between RBC life span and Epo requirement.
